# Thyroid hormone levels and temperature during development alter thermal tolerance and energetics of *Xenopus laevis* larvae

**DOI:** 10.1093/conphys/coy059

**Published:** 2018-11-17

**Authors:** Katharina Ruthsatz, Kathrin H Dausmann, Myron A Peck, Claudia Drees, Nikita M Sabatino, Laura I Becker, Janica Reese, Lisa Hartmann, Julian Glos

**Affiliations:** 1 Institute for Zoology, University of Hamburg, Martin-Luther-King-Platz 3, Hamburg, Germany; 2 Institute of Hydrobiology and Fisheries Science, University of Hamburg, Olbersweg 24, Hamburg, Germany; 3 Department of Life Sciences, Hamburg University of Applied Sciences, Ulmenliet 20, Hamburg, Germany

**Keywords:** Climate change, metabolic costs, metamorphosis, standard metabolic rate (SMR), thermal tolerance

## Abstract

Environmental variation induced by natural and anthropogenic processes including climate change may threaten species by causing environmental stress. Anuran larvae experiencing environmental stress may display altered thyroid hormone (TH) status with potential implications for physiological traits. Therefore, any capacity to adapt to environmental changes through plastic responses provides a key to determining species vulnerability to environmental variation. We investigated whether developmental temperature (*T*_dev_), altered TH levels and whether the interactive effect of both affect standard metabolic rate (SMR), body condition (BC), survival and thermal tolerance in larvae of the African clawed frog (*Xenopus laevis*) reared at five temperatures with experimentally altered TH levels. At metamorphosis, SMR, BC and survival were significantly affected by *T*_dev_, TH status and their interaction with the latter often intensified impacts. Larvae developing at warmer temperatures exhibited significantly higher SMRs and BC was reduced at warm *T*_dev_ and high TH levels suggesting decreased ability to acclimate to variation in temperature. Accordingly, tadpoles that developed at warm temperatures had higher maximum thermal limits but more narrow thermal tolerance windows. High and low TH levels decreased and increased upper thermal limits, respectively. Thus, when experiencing both warmer temperatures and environmental stress, larvae may be less able to compensate for changes in *T*_dev_. Our results demonstrate that physiological traits in larvae of *X. laevis* are strongly affected by increased TH levels and warmer temperatures. Altered TH levels and increasing *T*_dev_ due to global change may result in a reduced capacity for physiological plasticity. This has far reaching consequences since the energetic requirement at the onset of metamorphosis is known to determine metamorphic success and thus, is indirectly linked to individual fitness in later life stages.

## Teaser

Altered thyroid hormone levels as caused by environmental stress, developmental temperature and their interaction reduce the potential for physiological acclimation during metamorphosis in *Xenopus laevis*.

## Introduction

Environmental variation exposes wildlife to multiple chemical, physical and biological stressors that arise partly from anthropogenic activity (e.g. climate change, pollution), but also from natural sources (Noyes *et al.*, 2009). Many environmental stressors have the ability to impair endocrine function in wildlife (Carr and Patiño, 2011). Stressors which alter or disturb endocrine systems are characterized as endocrine disruptors (EDs) (Kloas and Lutz, 2006; Kloas *et al.*, 2009). The impact of EDs in the environment is of special concern in amphibians, which are the most threatened class of vertebrates on the planet (Stuart *et al.*, 2004; Hayes *et al.*, 2006; Gabor *et al.*, 2018). Particularly vulnerable are larval stages of amphibians due to the inability of this life stage to select or avoid habitats (Sanzo and Hecnar, 2006; Yu *et al.*, 2013) and their increased risk of exposure to chemical contaminants due to their highly permeable skin (Hayes *et al.*, 2006; Strong *et al.*, 2017). Furthermore, amphibian larvae are particularly sensitive to EDs since metamorphosis is linked to a reorganization of several organ systems, and this complex change underlies complicated and tight hormonal control (Hayes *et al.*, 2010; Searcy *et al.*, 2015).

Amphibian metamorphosis is a crucial event in amphibian life history due to the complex reorganization of larval to juvenile structures which is mainly regulated by thyroid hormones (TH) (i.e. T3 and T4) (Tata, 2006; Furlow and Neff, 2006). THs increase in concentration during metamorphosis and determine the developmental rate (Brown and Cai, 2007; Shi, 2000). Many EDs influence the hypothalamus–pituitary–thyroid axis, which is responsible for production of THs (Carr and Patiño, 2011). A large number of aquatic contaminants such as pesticides and herbicides, road salt, fertilizers, heavy metals and active pharmaceutical ingredients have been shown to disrupt and inhibit the normal action of THs in amphibians, leading to changes in growth, development and metabolism (Kashiwag *et al.*, 2009; Carr and Patiño, 2011). Inhibition or a decrease of TH production pathways slows the rate of development (Carr *et al.*, 2003; Bulaeva *et al.*, 2015) and decreases metabolic rates (Carr and Patiño, 2011; Ortiz-Santaliestra and Sparling, 2007) causing tadpoles to metamorphose at a larger size and older age (Shi, 2000). An environmentally relevant ED is perchlorate (ClO_4_^−^) which is a goitrogen that inhibits TH synthesis (Ortiz-Santaliestra and Sparling, 2007). Concentrations of perchlorate measured in the field are often high enough to inhibit amphibian metamorphosis (Goleman *et al.*, 2002; Ortiz-Santaliestra and Sparling, 2007; Tietge *et al.*, 2005).

Whereas most environmental contaminants inhibit TH activity or production pathways, some contaminants and other abiotic and biotic environmental factors appear to enhance TH activity or increase TH levels by the activation of the neuroendocrine stress axis (Mann *et al.*, 2009; Dantzer *et al.*, 2014) and increase of stress hormone levels (Denver, 1997). These stress hormones may lead to a synergistic increase in TH production (Glennemeier and Denver, 2002; Laudet, 2011; Kulkarni and Buchholz, 2012). The presence of predators (Relyea, 2002; Capellán and Nicieza, 2007), crowding (Morey and Reznick, 2001), desiccation risk (Gervasi and Foufopoulos, 2008), food scarcity (Kupferberg, 1997) and extreme temperature (Smith-Gill and Berven, 1979) may increase TH production by activating the neuroendocrine stress axis. Anuran larvae with high TH levels display increased developmental and metabolic rates and decreased growth rates (Rowe *et al.*, 1998; Brown and Cai, 2007), which results in shorter larval periods, smaller size at the onset of metamorphosis and higher energetic maintenance costs in addition to energetic developmental costs (Denver *et al.*, 1998; Denver, 2009; Orlofske and Hopkins, 2009). In this study, we simulated ecological stress by exposing larvae to L-thyroxin for an increase in TH levels and by inhibiting TH production by the environmental relevant ED sodium perchlorate.

In all vertebrates, THs are not only critical for regulating growth and development, but also for regulating energy metabolism (Sheridan, 1994; Choi *et al.*, 2017). If the TH concentration changes due to environmental stress, a whole suite of physiological processes may be affected. (Steyermark *et al.*, 2005; Hulbert and Else, 2004). Even if the effect of THs on metabolic heat production in ectotherms such as amphibians is negligible (John-Alder, 1983), THs increase the standard metabolic rate (SMR) which is estimated by measuring rates of O_2_ consumption at rest and represents the energy required to cover basic physiological functions (Rowe *et al.*, 1998; Beck and Congdon, 2003). In amphibians, elevated SMR due to increased TH level manifests in increased activities of enzymes and densities of mitochondria in metabolic relevant tissues such as liver and red skeletal muscle (Chiu and Woo, 1988; Rowe *et al.*, 1998; Steyermark *et al.*, 2005). In individuals with a low SMR but not reduced metabolic scope (the difference between active metabolic rate and SMR), more energy is available for physical performance or development (Steyermark *et al.*, 2005; Orlofske and Hopkins, 2009). As metamorphosis is an energy-consuming process (Sheridan and Kao, 1998; Beck and Congdon, 2003), it may be advantageous to maintain a low SMR. Tadpoles which have larger energy reserves at the onset of metamorphosis are more likely to successfully complete metamorphosis and become juvenile froglets with larger energy stores and higher rates of survival (Orlofske and Hopkins, 2009). Therefore, the SMR and body condition at the onset and after completion of metamorphosis are important fitness variables (Steyermark *et al.*, 2005; Muir *et al.*, 2014; Ruthsatz *et al.*, 2018).

Through its impact on physiology, temperature is considered to be the ‘abiotic master factor’ for ectotherms (Dalvi *et al.*, 2009; Turriago *et al.*, 2015; Sunday *et al.*, 2014; Berg *et al.*, 2017; Theisinger *et al.*, 2017). The tolerable thermal window of ectotherms is bracketed by critical temperatures (CT_min_ and CT_max_) beyond which survival is not possible and these limits occur where aerobic scope is either zero or negative (Holzman and McManus, 1973). Therefore, environmental factors that either load (increase) or unload (decrease) SMR will impact aerobic scope and, thus, the width of tolerable thermal windows (Burraco and Gomez-Mestre, 2016). Climate change is expected to not only result in long-term warming of aquatic habitats but also increased variability in temperature leading to new thermal challenges for tadpoles in their larval habitats (Gutiérrez‐Pesquera *et al.*, 2016) with likely impacts on growth, development and survival (Pörtner *et al.*, 2001; Dalvi *et al.*, 2009). Altered TH levels, through their impact on SMR, may exacerbate these thermal challenges experienced prior to and at the onset of metamorphosis (Formicki *et al.*, 2003). As THs have recently been shown to play a key regulatory role in thermal acclimation in fish (Little and Seebacher, 2014; 2016) and several studies provide an indication on thyroid-regulated acclimation in amphibians and reptiles (Locker and Weish, 1966; Packard and Packard, 1975; Little and Seebacher, 2016).

Although previous studies have examined the impact of stress-induced alteration of TH levels on metamorphic and physiological traits of anuran larvae, studies have rarely examined the interaction of different stressors which is known to affect amphibian metamorphosis under natural conditions (Rowe *et al.*, 1998; Freitag *et al.*, 2017). This study examined the interactive effects of temperature and altered TH levels on the capacity for physiological acclimation (SMR and thermal tolerance) at the onset of metamorphosis in larvae of *Xenopus laevis.* For larvae acclimated to five different temperatures and experimentally enhanced or lowered TH levels, we tested the following hypotheses: (i) High and low levels of TH, as caused by the thyroid altering effect of several environmental stressors, increase and decrease SMR of tadpoles, respectively. (ii) Changes in TH will be reflected in changes in body condition and survival. (iii) Developmental temperature (*T*_dev_) correlates positively with CT_min_, CT_max_, and negatively with the thermal range of tolerance. (iv) *T*_dev_ will interact with altered TH levels to intensify the effect on larval physiological traits.

## Material and methods

### Animal husbandry and experimental design

Three, unrelated clutches of *X. laevis* were obtained from the captive breeding facility of the Universitätsklinikum Hamburg Eppendorf (Martinistr. 52, 20 246 Hamburg, Germany). Larvae were allowed to develop to Gosner stage 25 (free-swimming larvae; Gosner, 1960). The experiment consisted of three replicate aquaria at each of three treatments and five temperatures. From these larvae, 675 individuals originating from different families were intermixed before allocating them randomly to 45 aquaria. Fifteen larvae of *X. laevis* were kept each in a standard 9.5-L aquarium filled with 8 L of water. The experiment was conducted in two controlled climate chambers (Weiss Umwelttechnik GmbH, 35 447 Reiskirchen, Germany) with a light regime of 12:12 L:D. The mean (±SD) water temperatures were 16 (±0.4), 19 (±0.5), 22 (±0.1), 25 (±0.5) and 28 (±0.3)°C. Temperature was maintained using ambient chamber temperature or via heaters and waterbaths. The experiments were conducted over 4 weeks, until all surviving larvae had reached the onset of metamorphosis (Gosner stage 42; Gosner, 1960). Amphibian larvae were fed high-protein flaked food (Sera, 52 518 Heinsberg, Germany) and spirulina algae *ad libitum*. The flakes were free of perchlorate according to the manufacturer. Dead or abnormal tadpoles were removed each day. All temperature measurements were made using a digital thermometer (Amarell, Maxi-Pen, −50 to 200°C: 0.1°C). All applicable international, national and/or institutional guidelines for the care and use of animals were followed. The experiments were approved by the *Amt für Verbraucherschutz, Lebensmittelsicherheit und Veterinärwesen* in Hamburg, Germany (Billstraße 80, 20 539 Hamburg, Germany; Gz. V1305/591–00.33, Nr. 03/16).

### Thyroxine and sodium perchlorate exposures

We used a concentration of 250 μg/L SP (Sodium perchlorate hydrate 99.99% trace metals basis, 381 225 Aldrich, Sigma-Aldrich, St. Louis, MO, USA) to achieve a decrease in TH levels (Tietge *et al.*, 2005). This concentration of SP is within environmental ranges measured in the surface and ground waters of many industrial nations (Motzer, 2001; Tietge *et al.*, 2005; Carr & Theodorakis, 2006; Mukhi and Patiño, 2007) and in bodies of water in which amphibians breed (Smith *et al.*, 2001; Ortiz-Santaliestra & Sparling, 2007).

We achieved increased TH levels by exposing tadpoles to 10 μg/L exogenous T4 (Thyroxine T4, IRMM468 Sigma-Aldrich, Sigma-Aldrich, St. Louis, MO, USA), a concentration which is known to influence amphibian metamorphosis (Lucas & Reynolds, 1967; Mann *et al.*, 2009) and is related to increases in T4 observed in tadpoles responding to stress (Denver, 1997; Denver *et al.*, 1998). Tadpoles absorb exogenous T4 directly through their permeable skin (Shi, 2000; Tata, 2006; Coady *et al.*, 2010).

T4 and SP treatments were prepared in 0.1 N sodium hydroxide solutions (Sodium hydroxide solution 0.1 N, S2770 SIGMA, Sigma-Aldrich, St. Louis, MO, USA) buffered with 0.1 N muriatic acid solutions as solvents. A clean solution of 0.1 M sodium hydroxide solution buffered with 0.1 M muriatic acid solution was added to the control aquaria to control for any effect of solvents addition. Each treatment and the control set-up was replicated three times (i.e. 45 larvae, 15 larvae per aquarium, per treatment and control in total). Water was changed every second day and fresh SP and T4 were added, which is frequent enough to maintain a constant hormone and perchlorate level under given experimental temperatures, in accordance with the standard procedure for chemical and hormonal addition (Miwa & Inui, 1987; Goleman *et al.*, 2002; Iwamuro *et al.*, 2003; Rot-Nikcevic & Wassersug, 2004; Tietge *et al.* 2005; Ortiz-Santaliestra & Sparling, 2007; Bulaeva *et al.*, 2015).

### Processing of specimens

Developmental stage was determined by evaluating the status of key morphological features typical of specific developmental stages, as detailed in Gosner (1960). The developmental stage of each tadpole was recorded according to the procedure of Ortiz-Santaliestra & Sparling (2007): Gosner stage groups 1–5: 1. pre-limb (absence of hind limbs, Gosner stages 24–26); 2. limb bud (hind limb visible, but no clear joint formed, Gosner stages 27–34); 3. middle hind limb (knee joint apparent, but toes not completely separated, Gosner stages 35–37); 4. late hind limbs (hind limb tubercles and subarticular patches formed, Gosner stages 38–41); and 5. metamorph (at least one forelimb present, Gosner stage 42 and above) (Gosner, 1960; Ortiz-Santaliestra & Sparling, 2007). The onset of metamorphosis was defined by the emergence of at least one forelimb (Gosner stage 42). The snout vent length (SVL) and total length of each larva was measured with a caliper to the nearest 0.5 mm. Larvae were blotted and weighed to the nearest 0.001 g with an electronic balance (digital gold scale, Smart Weigh). At the end of the experiment tadpoles were euthanized with 200 mg/L of tricaine methanesulfonate ([MS-222], Ethyl 3-aminobenzoate methanesulfonate, E10521 ALDRICH, Sigma-Aldrich, St. Louis, MO, USA) buffered with 200 mg/L of sodium bicarbonate (Sodium bicarbonate, S5761 SIGMA, Sigma-Aldrich, St. Louis, MO, USA) (Stuart *et al.*, 2007) and transferred into ethanol (70%).

### Body condition

We estimated the body condition (i.e. energy stores) at the onset of metamorphosis by calculating the scaled mass index (SMI). This is a measure of the entire body condition of an individual as it accounts for the allometric relationship between mass and a body structure measure. It standardizes each measure so that direct comparisons among individuals can be made (Peig & Green, 2009; 2010; MacCracken & Stebbings, 2012). The SMI was considered as an accurate condition index in anuran larvae (MacCracken & Stebbings, 2012; Dittrich *et al.*, 2016; Ruthsatz *et al.*, 2018). A high BC suggests larger energy storages and thus, a good body condition. We followed the procedure outlined by Peig and Green (2009) to calculate the SMI for each individual.

### Respiration measurements

Respiration measurements were made at the onset of metamorphosis on 45 individuals, three randomly chosen tadpoles from each aquarium. Animals were not fed for 48 h prior to and during the measurement of SMR such that tadpoles were in a post-absorptive state (Orlofske *et al.*, 2017). Oxygen consumption was measured by closed respirometry conducted between 0900 and 2100 h to control for the influence of natural circadian rhythms on respiration (Orlofske *et al.*, 2017). Larvae were placed in respirometers consisting of 30-mL beakers containing 30 mL (minus the volume of the animals) of autoclaved tap water to exclude microbial oxygen consumption. The water was at 100% O_2_ saturation at the start. Each respirometer was equipped with a fiber optic sensor (Oxygen Dipping Probe DP-PSt7; PreSens Precision Sensing GmbH, Regensburg, Germany) connected to a multichannel oxygen measuring system (Oxy 4 mini; PreSens Precision Sensing GmbH, Regensburg, Germany) and sealed with an airtight rubber plug. O_2_ concentration was recorded every 15 s and measured as ml O_2_ × L^−1^. Measurements were conducted at the developmental temperature of the individual. Prior to each trial, fiber optic O_2_ sensors were calibrated using air-saturated water and a factory-set zero oxygen calibration point at the respective measurement temperature. Water temperature was maintained by the continuous mixing of the waterbath. Oxygen consumption was measured for every tadpole for 20 min at each of five temperatures. Empty (control) chambers were run simultaneously in every trial and values were adjusted accordingly. We took care that <10% of total O_2_ was removed during the measurements to avoid impediment of respiration at low saturation levels. At the end of the measurements, each larva was removed and its TL, SVL and blotted wet body mass was determined.

### SMR calculations

Prior to statistical analysis, we plotted the O_2_ consumption rate of each tadpole over time and visually assessed activity peaks to exclude them for the determination of SMR (Orlofske and Hopkins, 2009). The SMR was expressed in ml O_2_ × h^−1^ × mg^−1^ wet body mass and was determined from the slope of linear least squares regressions of O_2_ concentration vs. time (Hastings and Burggren, 1995; Rowe and Funck, 2017).

Values for SMR and mass were log transformed because metabolism is a power function of mass (Orlofske and Hopkins, 2009; Orlofske *et al.*, 2017). To exclude the mass-specific effect (Hulbert and Else, 2004) on SMR we did a linear regression of log transformed mass and log transformed SMR to calculate residuals. Residuals obtained from this regression (SMR_residuals_) were entered into the analyses instead of SMR. We performed a multiple linear regression of log transformed SMR (dependent variable), log transformed mass (independent variable) and developmental temperature (independent) to describe the mass and temperature dependence of SMR.

### Thermal tolerance

Thermal tolerance of *X. laevis* was evaluated when tadpoles reached the onset of metamorphosis (Gosner stage 42) using the critical thermal methodology (CTM) (Holzman and McManus, 1973). Both critical thermal maximum (CT_max_) and minimum (CT_min_) endpoints are defined as the thermal point at which locomotor activity becomes disorganized and the animal loses the ability to right itself (Lutterschmidt and Hutchison, 1997; Turriago *et al.*, 2015). A total of 90 tadpoles were used for determination of thermal tolerance. From each aquarium six tadpoles (*n* = 3, CT_max_; and *n* = 3, CT_min_) were tested at set time intervals. CT_min_ and CT_max_ were determined by using the dynamic method according to Cowles and Bogert (1944) and Hutchison (1961) except for the end point (Wu and Kam, 2005). This method involves increasing (for CT_max_) or decreasing (for CT_min_) test temperatures by a specific rate until an appropriate end point is reached (Lutterschmidt and Hutchison, 1997). Tadpoles were placed individually in a 250-ml flask with 200 ml of water which was then placed in a temperature-controlled waterbath. The heating and cooling rates were ±0.1°C × min^−1^, and the water temperature served as a proxy of body temperature (Hutchison, 1961). The initial temperature in the waterbath was set at the respective developmental temperature. In tadpoles, the occurrence of spasms is difficult to determine, and thus we decided to use the loss of the righting response after being flipped on its back in the water with a probe as our criterion for the end point (Lutterschmidt and Hutchison, 1997; Wu and Kam, 2005) for both, CT_min_ and CT_max_ determinations (Turriago *et al.*, 2015). A time limit of 30 s between flipping the animal and righting was adopted (Layne and Claussen, 1982). All thermal tolerance tests were performed between 1100 and 1500 h. After the experiments, we euthanized the tadpoles with MS-222, weighed them and measured SVL and TL, and finally transferred them into ethanol (70%).

We adopted the method of Dalvi *et al.* (2009) used in fish to generate a thermal tolerance window (TW) for *X. laevis* by calculating the difference between CT_max_ and CT_min_ estimates obtained at various acclimation temperatures. To simulate long-term changes in environmental temperature, *X. laevis* were reared at a range of different temperatures. The thermal tolerance polygon was generated by plotting the five developmental temperatures for each treatment (T4, SP and Control) on the *X*-axis and the mean CT_min_ and CT_max_ values on the *Y*-axis. The TW was calculated from the polygon and expressed as °C^2^ (Dalvi *et al.*, 2009). We performed a linear regression for developmental temperature and thermal tolerance (as measured by CT_min_, CT_max_ and thermal range of tolerance). The slope of the regression for CT_max_ and CT_min_ defined the effect of developmental temperature on critical thermal limits of *X. laevis*.

### Statistical analyses

For all statistical tests R 3.4.1 (R Development Core Team, 2007) for Windows was used. All plots were constructed using ggplot2 (Wickham 2009) and Adobe Illustrator CS6. Data were analyzed using linear mixed-effect models [lme, Type III model, covariance type: variance components, REML (restricted maximum likelihood) method for parameter estimation, 100 iterations (Bates and Sarkar, 2007)], using the covariate ‘T_dev_’, and ‘treatment’ (T4, SP and Control) and the interactions of ‘treatment’ and ‘*T*_dev_’ as fixed factors. ‘SMR_residuals_’, ‘body condition’, ‘survival’ and ‘thermal tolerance’ (as measured by CT_min_, CT_max_ and the thermal range of tolerance) were used as dependent variables in separate models. *P*-values were obtained from likelihood-ratio tests (Crawley, 2012). To address dependencies in the data, the variable ‘aquarium’ was included as a random factor. Residuals of each model were visually checked for normal distribution. *N* refers to the total number of analyzed tadpoles. Linear mixed-effect models were followed by post hoc comparisons (Tukey’s test; Tukey HSP function, multcomp package, vers. 1.2–13) to compare all possible pairwise combinations of treatments when overall tests were significant.

‘Thermal tolerance’ (as measured by CT_min_, CT_max_ and the thermal range of tolerance) was correlated with SMR and *T*_dev_ using Spearman’s rank correlation (Table 1). Correlations were performed on subgroups according to TH-treatment and Control. The slope of the linear regression for CT_max_ and CT_min_ with *T*_dev_ defined the effect of temperature during development on the thermal tolerance of *X. laevis* (Table 1) (Dalvi *et al.*, 2009).

**Table 1:** Effects of altered TH levels and temperature during development on SMR, body condition, survival and thermal tolerance (as measured by CT_min_, CT_max_ and thermal range of tolerance) in tadpoles of the African clawed frog (*X. laevis*) at the onset of metamorphosis (Gosner stage 42) (Gosner, 1960). Chi^2^ and *P* for linear mixed-effects models, using the covariates ‘Treatment’ (T4, SP and Control), ‘*T*_dev_’ and the interactions of ‘Treatment*T_dev_’ as fixed factors; ‘aquarium’ as random factor. T4 = increased TH concentration; SP = decreased TH concentration. Significance was set at *P* < 0.05. SMR was taken as the residuals from the regression of log transformed mass on log transformed SMR. Body condition was determined by the scaled mass index (SMI). Bold for significant values

Dependent variable	Fixed effects	*Xenopus laevis*					
		Estimate	SE	Chi^2^	Df	*P*	*N* (*n*)
SMR_residuals_	Treatment [Control]	−0.08	0.04	39.63	2	**<0.001**	119 (9)
	Treatment [SP]	−0.10	0.08	39.63	2	**<0.001**	119 (9)
	Treatment [T4]	0.13	0.06	39.63	2	**<0.001**	119 (9)
	*T*_dev_	−0.08	0.06	39.63	1	**<0.001**	119 (9)
	*T*_dev_* Treatment [SP]	−0.15	0.09	39.63	2	**<0.001**	119 (9)
	*T*_dev_* Treatment [T4]	0.33	0.09	39.63	2	**<0.001**	119 (9)
Body condition (SMI)	Treatment [Control]	275.56	36.11	11.42	2	**0.003**	475 (45)
	Treatment [SP]	69.48	53.23	11.42	2	**0.003**	475 (45)
	Treatment [T4]	−156.08	55.72	11.42	2	**0.003**	475 (45)
	*T*_dev_	−3.98	1.61	11.42	1	**0.003**	475 (45)
	*T*_dev_* Treatment [SP]	−3.09	2.36	11.42	2	**0.003**	475 (45)
	*T*_dev_* Treatment [T4]	−5.52	2.49	11.42	2	**0.003**	475 (45)
Survival (%)	Treatment [Control]	−0.23	0.11	11.05	2	**0.003**	(45)
	Treatment [SP]	0.32	0.16	11.05	2	**0.003**	(45)
	Treatment [T4]	0.61	0.16	11.05	2	**0.003**	(45)
	*T*_dev_	0.01	0.01	11.05	1	**0.003**	(45)
	*T*_dev_* Treatment [SP]	−0.01	0.01	11.05	2	**0.003**	(45)
	*T*_dev_* Treatment [T4]	−0.02	0.01	11.05	2	**0.003**	(45)
CT_min_ (°C)	Treatment [Control]	−11.26	0.96	37.02	2	**<0.001**	135 (45)
	Treatment [SP]	0.76	1.35	37.02	2	**<0.001**	135 (45)
	Treatment [T4]	0.39	1.35	37.02	2	**<0.001**	135 (45)
	*T*_dev_	0.99	0.04	343.16	1	**<0.001**	135 (45)
	*T*_dev_* Treatment [SP]	−0.01	0.06	1.41	2	0.49	135 (45)
	*T*_dev_* Treatment [T4]	−0.01	0.06	1.41	2	0.49	135 (45)
CT_max_ (°C)	Treatment [Control]	28.15	2.23	8.59	2	**0.01**	135 (45)
	Treatment [SP]	1.64	3.16	8.59	2	**0.01**	135 (45)
	Treatment [T4]	−0.7	3.16	8.59	2	**0.01**	135 (45)
	*T*_dev_	−0.28	0.09	24.02	1	**<0.001**	135 (45)
	*T*_dev_* Treatment [SP]	−0.02	0.14	0.05	2	0.97	135 (45)
	*T*_dev_* Treatment [T4]	−0.00	0.14	0.05	2	0.97	135 (45)
Thermal range of tolerance	Treatment [Control]	39.41	1.73	0.89	2	0.63	135 (45)
	Treatment [SP]	0.87	2.44	0.89	2	0.63	135 (45)
	Treatment [T4]	−1.09	2.44	0.89	2	0.63	135 (45)
	*T*_dev_	−0.69	0.07	138.08	1	**<0.001**	135 (45)
	*T*_dev_* Treatment [SP]	−0.02	0.1	0.68	2	0.71	135 (45)
	*T*_dev_* Treatment [T4]	0.06	0.1	0.68	2	0.71	135 (45)

## Results

### Standard metabolic rate

**Figure 1:**
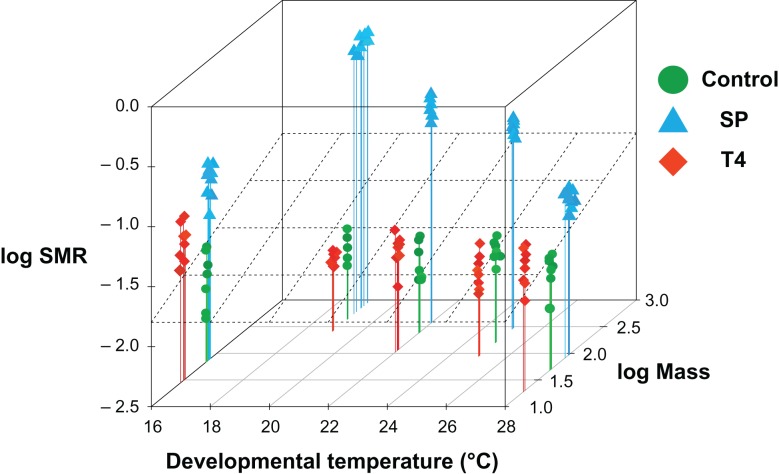
Mass and temperature dependence of standard metabolic rate (SMR) in *Xenopus laevis*. Multiple linear regression of SMR (ml O_2_ × h^−1^ × mg^−1^) on developmental temperature and mass (mg; log transformed) at in various experimental treatments in *X. laevis* in a total of 119 animals. Gray lines and dots: control animals. Orange lines and triangle: Low thyroid hormone levels (SP treatment). Blue lines and squares: High thyroid hormone levels (T4 treatment). Dotted plane: Average regression plane for multiple linear regressions including all treatments: log SMR(Control) =−1.62488 + (−0.21295) × log Mass + (0.01217) × *T*_dev_; log SMR(T4) = −0.2795 + (−0.4918) × log Mass + (−0.0201) × *T*_dev_; log SMR(SP) =−2.06228 + (0.74817) × log Mass + (−0.02252) × *T*_dev_.

SMR at *T*_dev_ of tadpoles was significantly influenced by the hormone treatment, by *T*_dev_ and the interactive effect of both (Table 1). There was no consistent effect of time of day on SMR tested during this experiment. At all developmental temperatures tadpoles from the SP treatment were the largest at the onset of metamorphosis followed by control animals and tadpoles from the T4 treatment being the smallest (Table A1 and Fig. 1). Mass-specific SMR decreased with increasing mass (Fig. 1) with the highest SMR observed in tadpoles from the T4 treatment followed by individuals in the SP treatment. The lowest SMR was observed in the largest tadpoles (SP treatment), followed by control group animals, and T4 treated animals (Fig. 1). At all developmental temperatures, tadpoles exposed to T4 had the highest SMR.

### Body condition and survival

**Figure 2:**
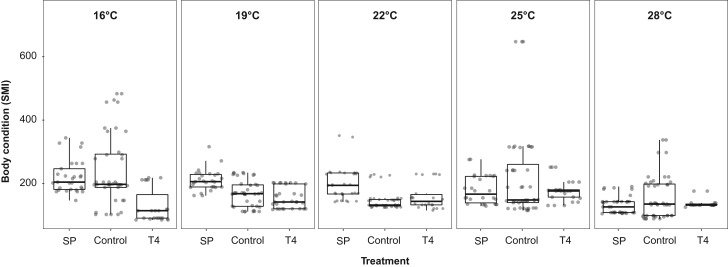
Interactive effect of altered thyroid hormone levels and five developmental temperatures on body condition in tadpoles of the African clawed frog (*X. laevis*) at the onset of metamorphosis (Gosner stage 42) (Gosner, 1960). Body condition was determined by the scaled mass index (SMI).

Body condition of *X. laevis* was significantly affected by treatment, temperature during development, as well as by the interactive effect of both (Table 1 and Fig. 2). High TH levels, *T*_dev_ and the interactive effect of altered TH levels and *T*_dev_ lead to a reduced body condition and thus, energy stores in tadpoles of *X. laevis* whereas low TH levels increased body condition.

Survival of *X. laevis* was significantly affected by hormone treatment, developmental temperature, as well as by the interactive effect of both (Table 1). Mean (±SD) survival from the start of the experiment (Gosner stage 25) to the onset of metamorphosis (Gosner stage 42) in the Control, SP and T4 treatment groups was 92.0 (±10.0), 65.7 (±13.3) and 53.3 (±13.3) %, respectively. High levels of TH reduced survival to nearly half of that observed in the Control. This effect was intensified by the interactive effect of TH level and temperature. Larvae from 19°C—Control, 25°C—SP and 28°C—T4 treatments/groups revealed the lowest survival among all *X. laevis* tadpoles (Table A2). Most larvae survived in 25°C—Control, 28°C—SP and 19°C—T4 treatments/groups (Table A2).

### Thermal tolerance and thermal window

There were significant effects of *T*_dev_ on CT_min_, CT_max_ and the thermal range of tolerance within all three treatment groups (Table 1). There was no interactive effect of altered TH status and temperature during development on CT_min_, CT_max_ and the thermal range of tolerance. Whereas TH status affected CT_min_ and CT_max_, there was no effect on the thermal range of tolerance. CT_max_ was reduced at high TH levels and increased at low TH levels, whereas low TH levels increased both CT_min_ and CT_max_. The thermal range of tolerance was only affected by temperature during development.

The CT_min_ and CT_max_ increased significantly with increasing developmental temperature, whereas the thermal range of tolerance decreased with increasing developmental temperature (Table A3). The SMR did not correlate with CT_min_, CT_max_ or the thermal range of tolerance (Table A3).

The regression slope for CT_min_ and CT_max_ of *X. laevis* shows that for every 1°C increase in *T*_dev_ the CT_min_ and CT_max_ increased by 0.98 and 0.31°C, respectively, in the control group, by 0.96 and 0.28°C in the T4 treatment, and by 0.95 and 0.27°C in the SP treatment (Table A4). In all treatments and control group developmental temperature had a greater effect on CT_min_ than on CT_max_ (Table A4).

**Figure 3:**
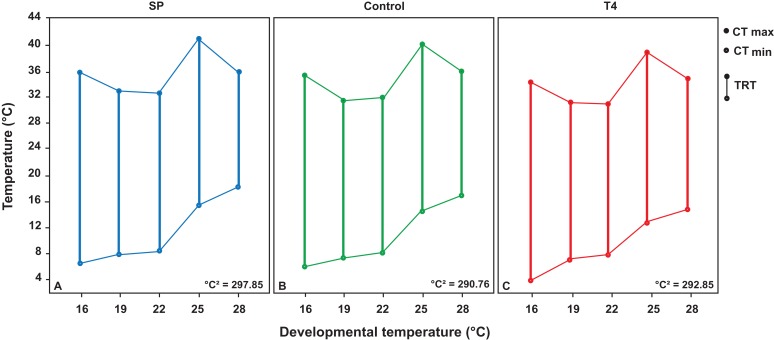
Developmental thermal windows of *X. laevis*. Thermal tolerance polygons generated from the critical thermal limits (CT_min_ and CT_max_) at five developmental temperatures in a total of 45 animals. (**A**) Control animals. (**B**) Low thyroid hormone levels (SP treatment). (**C**) High thyroid hormone levels (T4 treatment).

The thermal window polygon area (TW) for *X. laevis* reared at five different temperatures between 16 and 28°C was calculated as 290.76°C^2^ for the control group, 297.86°C^2^ for the SP treatment and 292.87°C^2^ for the T4 treatment (Fig. 3 and Table 1).

## Discussion

Aquatic organisms such as anuran larvae are limited in their ability to search for favorable microhabitats (Gutiérrez‐Pesquera *et al.*, 2016). Therefore, knowing the capacity of species to react to environmental change through plastic responses is a key to determining how vulnerable species with limited mobility will be to environmental variation and global climate change (Martinez *et al.*, 2016; Gutiérrez‐Pesquera *et al.*, 2016; Berg *et al.*, 2017). Species that cannot compensate for long-term (e.g. average warming) or short-term (e.g. increased variability) changes in abiotic factors by buffering metamorphic and physiological traits will be most affected (Kern *et al.*, 2015). Therefore, we investigated whether *T*_dev_, altered TH levels, and the interactive effect of both affect physiological traits in larvae of *X. laevis* as a model system and determined their capacity for physiological plasticity. Our results demonstrate that physiological traits in larvae of *X. laevis* are strongly affected by increased TH levels and warmer developmental temperatures. Our results for *X. laevis* suggest that altered TH levels, which can result from various environmental stressors, and warmer developmental temperatures will result in a reduced capacity for physiological plasticity in anuran larvae.

### Altered TH levels and warmer temperatures increase SMR and affect body condition

Amphibians are ectothermic animals and ambient temperature regulates the rates of all physiological and biochemical processes (Smith-Gill and Berven, 1979; Tata, 2006; Little and Seebacher, 2016) impacting growth, development and metabolism. An increase in ambient temperature may, therefore, increase the SMR but animals may compensate for those thermal changes through acclimation (Angilletta *et al.*, 2006; Berg *et al.*, 2017). In this study, the SMR of larval *X. laevis* markedly increased at warmer temperatures suggesting limited capacity to compensate for warming through physiological acclimation.

As climate change is increasing mean environmental temperatures and the frequency of extreme thermal events (Pachauri *et al.*, 2014; Gutiérrez‐Pesquera *et al.*, 2016; Theisinger *et al.*, 2017), species with a limited capacity for acclimation will suffer from high maintenance costs as caused by the high SMRs. Especially in anuran larvae, having a low energy expenditure before and during metamorphosis is favorable since metamorphosis is a highly energy-consuming process (Orlofske and Hopkins, 2009). A high SMR before and at the onset of metamorphosis may reduce the ability of larvae to store energy (Sheridan and Kao, 1998; Orlofske and Hopkins, 2009; own unpublished data). Energy stores are used during metamorphic climax when larvae stop feeding due to the rebuilding of the gastro-intestinal tract and changes in oral morphology (Beck and Congdon, 2003; Orlofske and Hopkins, 2009). Depending on how much of the accumulated energy is needed for covering maintenance costs (i.e. high or low SMR), more or less of this stored energy is available for covering the costs of development (Steyermark *et al.*, 2005; Beck and Congdon, 2003; Orlofske and Hopkins, 2009) and, consequently, the larvae will have a higher or lower probability of successfully completing metamorphosis.

Anuran larvae which show limited capacity for an acclimation in SMR, such as *X. laevis* in the present study but especially tropical amphibians in general (Janzen, 1967), may be least able to tolerate additional, climate-driven warming of their habitat. Under natural conditions, any trait, such as increased SMR, that increases metamorphic rates and reduces the transition time through this vulnerable life history stage, would be preferentially selected. However, we observed reduced body condition (lower energy stores) in tadpoles reared at warmer temperatures and experiencing high levels of THs as caused by environmental stress. Environmental stress and global warming, thus, may result in increased SMRs which, in turn, impair the capacity to store energy needed for the complex reorganization during metamorphic climax. Metabolic acclimation (decreasing SMR) may be advantageous when the risk of desiccation and predation is low as it allows more energy to be accumulated and subsequently allocated to development during metamorphic climax. In addition to increases in mean environmental temperatures, altered TH levels frequently accompanies the thermal effects on developmental and metabolic rate in anuran larvae. In this study, larvae of *X. laevis* revealed a higher SMR when exposed to T4 and a reduced SMR when exposed to SP. This effect was intensified at warmer temperatures. Hence, our results confirmed that the level of TH determines the metabolic rate in *X. laevis* as shown for lizards (*Dipsosaurus dorsalis* and *Sceloporus occidentalis*; John-Alder, 1983; 1990), snakes (*Thamnophis sirtalis*; Etheridge, 1993) and the leopard frog (*Rana pipiens*; Steyermark *et al.*, 2005), but had been negated for juvenile *X. laevis* (Dupré *et al.*, 1986). Therefore, larvae exposed to warmer temperatures are likely to be affected more by environmental stressors than larvae at colder temperatures before and during metamorphic climax.

### Long-term temperature changes: developmental temperatures determine critical thermal limits , thermal range of tolerance and the thermal window

Apart from metabolic acclimation concomitant with a low sensitivity of SMR to short-term temperature variation, changes in thermal tolerance may provide a key mechanism to help amphibian larvae cope with the longer-term impacts of climate-driven warming and increased frequency of extreme environmental? events (Seebacher *et al.*, 2015; Gutiérrez‐Pesquera *et al.*, 2016). In this study, tadpoles from warm developmental temperatures had higher thermal limits, but a narrower thermal range of tolerance than tadpoles from colder treatments. Consequently, larvae of *X. laevis* have the ability to compensate for changes in developmental temperature as they increased their thermal limits at warmer developmental temperatures (Schaefer and Ryan, 2006; Gunderson and Stillman, 2015; Little and Seebacher, 2016). The driver for this adjustment are changes in the thermal reaction norm and hence of physiological nature (Little and Seebacher, 2016; Theisinger *et al.*, 2017).

Although our results demonstrate the ability of *X. laevis* tadpoles to acclimate to different temperatures by changing their critical thermal limits and thermal range of tolerance, the latter was narrower when tadpoles were raised at warmer temperatures. Those warm-acclimated larvae may be more vulnerable to the impacts of climate change in terms of lacking the capacity for an acclimation in other physiological traits (Gunderson and Stillman, 2015) as larvae in the present study were not able to acclimate their SMR to warmer temperatures.

We found a significanteffect of altered TH levels on the thermal limits in *X. laevis* indicating that the thyroid systems affects the capacity to acclimate to temperature variation. Therefore, altered TH levels as caused by environmental stress affect the capacity for an acclimation in thermal limits but not in the thermal range of tolerance. Accordingly, larvae under environmental stress are constrained in their ability to compensate for changes in developmental temperature. Furthermore, they might suffer from the consequences of high SMR on body condition and thus, energy stores.

## Conclusions

Considering the current worldwide decline of amphibians (Alroy, 2015; Stuart *et al.*, 2004) it is of major interest to investigate whether and how anuran larvae adjust their physiological traits to new thermal challenges and to altered TH status, as caused by natural or anthropogenic stressors in their larval habitat (Strong *et al.*, 2017). Increased metabolic rates are the expected future responses of ectotherms (Seebacher *et al.*, 2015; Berg *et al.*, 2017), especially in species with reduced physiological plasticity due to extreme but stable environments as common in tropical species (Janzen, 1967; Huey *et al.*, 2012; Oyamaguchi *et al.*, 2017). Even though *X. laevis* is often used for laboratory experiments and, thus, cultured under constant thermal conditions, this is a tropical (Sub-Saharan Africa) species adapted to warm temperatures. Our findings emphasize how environmental stress and climate-driven warming may be detrimental to tadpoles of *X. laevis* under natural conditions by causing limits to the acclimation of physiological traits leading to reduced body condition. The lack of comparative data on SMR at different temperatures and critical thermal limits necessitates future work to explore how environmental stress may influence physiological traits at the onset of metamorphosis in other species of amphibians. Comparative studies across populations and species would help to identify the potential for local adaptation or inter-specific differences, respectively, on physiological traits affecting the age and size at metamorphosis. Furthermore, as TH levels alter metabolism and body condition in both larval and adult amphibians (Tata *et al.*, 1962), environmental stress may affect froglets and frogs alike, having long-lasting effects on amphibian populations. Long-term studies are needed to understand the consequences of various stressors during larval stages on the phenotype and on the fitness of juveniles and the adults.

## Appendices

**Table A1.** Descriptive statistics of mass (mg) at the onset of metamorphosis in tadpoles of the African clawed frog *Xenopus laevis* at five different developmental temperatures exposed at different TH levels. T4 = high TH levels; SP = low TH levels

	Temperature	Treatment	Maximum	Minimum	Mean	SE	*N*
Mass (mg)	16	Control	68	74	71.27	0.312	40
		SP	73	84	77.76	0.574	29
		T4	28	33	31.26	0.399	23
	19	Control	410	452	442.64	1.190	39
		SP	551	897	695.07	18.186	27
		T4	259	275	267.87	0.698	31
	22	Control	234	254	243.37	0.930	41
		SP	369	381	375.45	0.681	31
		T4	100	119	110.45	1.309	22
	25	Control	152	164	159.89	0.455	44
		SP	284	303	296.12	1.144	26
		T4	85	95	89.88	0.441	25
	28	Control	45	54	48.81	0.357	43
		SP	82	96	93.14	0.600	35
		T4	19	21	20.11	0.105	19

**Table A2.** Survival (%)at the onset of metamorphosis in tadpoles of *Xenopus laevis* exposed to different combinations of developmental temperature (*T*_dev_) and altered TH levels. T4 = increased TH levels; SP = decreased TH levels

Group		Survival (%) ± SD
*T*_dev_	Treatment	*Xenopus laevis*
16	Control	91.11 ± 8.89
	T4	51.11 ± 2.22
	SP	66.67 ± 0
19	Control	86.67 ± 6.67
	T4	68.89 ± 4.44
	SP	60.6 ± 6.67
22	Control	91.11 ± 8.89
	T4	48.89 ± 4.44
	SP	68.89 ± 4.41
25	Control	97.78 ± 2.22
	T4	55.56 ± 4.44
	SP	57.78 ± 8.89
28	Control	93.33 ± 0
	T4	42.22 ± 4.45
	SP	75.56 ± 4.44

**Table A3.** Correlation of critical thermal limits (as measured by CT_min_ and CT_max_) and thermal range of tolerance with temperature during development and standard metabolic rate in *X. laevis* tadpoles. *ρ* (correlation coefficient) and *P* for Spearman’s rank correlation. Significance was set at *P* < 0.05. T4 = high TH levels; SP = low TH levels

Dependent variable	Treatment	Temperature during development (°C)		SMR (ml O__2__ × h^−1^ × mg^−1^)	
		*ρ*	*P*	*ρ*	*P*
CT_min_	Control	0.96	**<0.001**	0.09	0.54
	T4	0.96	**<0.001**	0.08	0.57
	SP	0.97	**<0.001**	0.03	0.84
CT_max_	Control	0.36	**0.014**	0.07	0.61
	T4	0.43	**0.003**	0.42	0.40
	SP	0.38	**0.009**	0.07	0.61
Thermal range of tolerance	Control	−0.66	**<0.001**	−0.14	0.34
	T4	−0.68	**<0.001**	0.08	0.57
	SP	−0.67	**<0.001**	−0.12	0.41

Bold indicates significant *P*-values.

**Table A4.** Critical thermal minima (CT_min_), critical thermal maxima (CT_max_), thermal range of tolerance and thermal window (TW) (±SD) of tadpoles of the African clawed frog *Xenopus laevis* at five different temperatures during development exposed at different TH levels. T4 = high TH levels; SP = low TH levels. Regression slopes show the increase of CT_min_ and CT_max_ for every 1°C increase in *T*_dev_. Bold for mean values

Developmental temperature (°C)	Treatment	CT_min_ (°C) ±SD	CT_max_ (°C) ±SD	Thermal range of tolerance ±SD	TW (°C^2^)	Regression slope CT_min_ (*R*^2^)	Regression slope CT_max_ (*R*^2^)
16	Control	**5.5** ± 0.4	**35.1** ± 0.8	**29.6** ± 0.4	290.76	Y = 0.98x−11.28 (0.891)	Y = 0.31x+28.04 (0.161)
19		**7.3** ± 0.1	**31.5** ± 0.5	**24.1** ± 0.3			
22		**8.4** ± 0.1	**31.6** ± 0.3	**23.1** ± 0.8			
25		**14.2** ± 0.2	**39.7** ± 0.2	**25.5** ± 0.5			
28		**16.9** ± 0.1	**35.5** ± 0.5	**18.5** ± 0.4			
16	SP	**4.2** ± 0.7	**34.3** ± 0.6	**30.1** ± 0.3	297.85	Y = 0.96x−10.66 (0.893)	Y = 0.28x+28.58 (0.122)
19		**7.1** ± 0.9	**30.9** ± 0.1	**23.8** ± 0.6			
22		**7.8** ± 0.1	**30.8** ± 0.1	**23** ± 0.5			
25		**13.1** ± 0.8	**39.0** ± 0.5	**25.8** ± 0.6			
28		**15.1** ± 0.4	**34.8** ± 0.1	**19.7** ± 0.7			
16	T4	**6.5** ± 0.5	**35.9** ± 1.1	**29.4** ± 1.1	292.875	Y = 0.95x−10.68 (0.907)	Y = 0.27x+28.77 (0.124)
19		**7.9** ± 0.5	**32.7** ± 0.2	**24.8** ± 0.1			
22		**8.5** ± 0.5	**33.1** ± 0.9	**24.5** ± 0.4			
25		**15** ± 0.5	**41.1** ± 0.3	**26.1** ± 0.3			
28		**17.7** ± 0.7	**35.8** ± 0.1	**18.1** ± 1.9			
